# Do we spend less on older critically ill patients? Relationship among intensity of care, severity of illness and mortality

**DOI:** 10.1186/cc13212

**Published:** 2014-03-17

**Authors:** MD Rosa, AC Pecanha Antonio, M Mattioni, L Tagliari, F Schaich, J Maccari, R Oliveira, C Teixeira, TF Tonietto, N Brandäo da Silva

**Affiliations:** 1 Hospital Moinhos de Vento, Porto Alegre, Brazil

## Introduction

The Therapeutic Intervention Scoring System (TISS-28) quantifies the type and number of intensive care treatments; therefore, it indicates the workload of ICU and may be used for calculating costs [1]. A previous cohort study [2] demonstrated that seriously ill older patients receive fewer invasive procedures and less resource-intensive hospital care, showing a preferential allocation of hospital services to younger patients regardless of severity of illness. The objective of this study is also to compare resource utilization across different ages of critically ill patients in an open model, mixed ICU.

## Methods

During 7 years (2007 to 2013), TISS-28 was prospectively applied to all consecutive adult critically ill patients at 24 and 72 hours of ICU admission in a private hospital in Brazil. Demographic data, diagnoses on admission, comorbidities, ICU length of stay and mortality were recorded. Patients were stratified according to their ages.

## Results

TISS-28 scores at 24 and 72 hours of ICU admission were analyzed for 4,128 patients. Mean patient age was 68.3 (SD ± 17.6), 46.8% were female and 37% were surgically ill. The mean APACHE II score was 16 (SD ± 8) and 40% were submitted to mechanical ventilation at any time of the stay. Overall mortality was 14.4%. Neither APACHE II score adjusted for age nor TISS-28 in 24 and 72 hours of admission differs among age groups. However, mortality was significantly higher in patients aged 70 years or older (*P *< 0.001).

## Conclusion

Mortality remained higher in older patients despite an absence of age-related differences in resource use at ICU. Comparing our results to a previous prospective cohort study [2], we emphasize the lower overall mortality of our population (14.4% vs. 50%). Limitations of our analysis include our unicenter design and lack of data for a closed model ICU.
